# Concomitant Polyoma BK Virus and West Nile Virus in Renal Allografts

**DOI:** 10.3390/pathogens12121456

**Published:** 2023-12-15

**Authors:** Riddhish T. Sheth, Dalia Y. Ibrahim, Amira F. Gohara, Obi Ekwenna, Michael A. Rees, Deepak Malhotra, William T. Gunning

**Affiliations:** 1 Department of Pathology, University of Toledo, Toledo, OH 43614, USA; riddhish.sheth@utoledo.edu (R.T.S.); dalia.ibrahim@utoledo.edu (D.Y.I.); amira.gohara@utoledo.edu (A.F.G.); 2 Department of Urology, University of Toledo, Toledo, OH 43614, USA; obinna.ekwenna@utoledo.edu (O.E.); michael.rees2@utoledo.edu (M.A.R.); 3 Department of Medicine, University of Toledo, Toledo, OH 43614, USA; deepak.malhotra@utoledo.edu

**Keywords:** kidney, allograft, transplant, BK, West Nile, virus, biopsy

## Abstract

Surveillance of the renal allograft recipient is essential when monitoring renal function to detect the early onset of rejection and alter therapeutic treatments to treat acute rejection or other causes and improve long-term graft function. If renal function begins to deteriorate, a renal biopsy is often indicated to assess the Banff grade of potential rejection or other causes, especially in the setting of polyoma BK viral load elevation. Although BK infection in the allograft is asymptomatic, reactivation of the virus is known to be associated with the acceleration of pathologic change and a poor outcome in the allograft. BK reactivation in a transplant kidney is not uncommon, and determining inflammation related to the virus versus acute rejection is paramount for appropriate immunosuppressive therapy management. We identified a concomitant polyoma BK virus and West Nile Virus (WNV) infection in two renal transplant patients which, to our knowledge, has not previously been reported. However, other concomitant infections have been reported in renal allografts including BK virus and cytomegalovirus (CMV), CMV and hepatitis C (HCV), and HCV and human immunodeficiency virus (HIV). As WNV has become endemic in many regions of the United States, and since the transmission of the virus via transplanted organs is associated with significant morbidity and mortality, it may be prudent to consider serologic screening for WNV in living donors prior to organ procurement. Regardless, the observation we made and report here should underscore the potential for concomitant viral infections that may be masked when a renal allograft has a significant inflammatory response to BK virus.

## 1. Introduction

An immunosuppressed state, such as in the post-renal-transplant patient, has many associated risks and complications, including susceptibility to the reactivation of latent viral infection such as BK virus or cytomegalovirus. BK polyomavirus is ubiquitous in humans, and its activation in an immunosuppressed renal transplant recipient may result in the elevation of creatinine, suggesting the potential for allograft rejection [1,2,3]. Cytomegalovirus (CMV) in an immunosuppressed patient can predispose the patient to renal allograft rejection and may also result in a varied spectrum of organ involvement ranging from respiratory tract and gastrointestinal infections, retinitis, and myelosuppression to neurological involvement [4]. Cytomegalovirus pneumonitis in renal transplant patients is a well-known complication with high mortality and morbidity rates [5,6]. Herpes simplex virus 1 and 2 (HSV) has been reported to reactivate in the post-renal-transplant immunocompromised state and lead to acute nephritis and potential allograft failure [7]. One study of 40 renal allograft patients identified HSV antibodies in 95% of the subjects prior to transplantation [8]. Adenovirus can cause hemorrhagic cystitis and tubulointerstitial nephritis in kidney transplant patients [9]. More recently, West Nile virus (WNV) infection has been reported to cause renal graft dysfunction and failure but may also produce a devastating viral encephalitis that is often fatal [10,11,12,13]. It has been established that WNV can be harbored in the kidney and remain dormant for years [14]. Infection with WNV is usually asymptomatic, but with the immunosuppressed state of a transplant recipient, an increased susceptibility to reactivation exists [11,15]. A number of reports have been published suggesting that WNV harbored in a solid organ donor, especially in the kidney, can become activated in an immunosuppressed host, leading to severe consequences [11,16].

We have discovered concomitant WNV infection in two renal allograft patients being evaluated for rejection versus the reactivation of BK virus; both patients had elevations of both creatinine and BK viral titers.

## 2. Materials and Methods

This retrospective study was exempt from Institutional Review Board Approval. Two male renal transplant patients, 37 and 61 years old, suspected of having allograft rejection or BK viral infection underwent percutaneous needle biopsy to obtain tissue for morphologic assessment via light, immunofluorescence, and electron microscopy (EM).

## 3. Results

Both patients had significant mononuclear cell infiltrates in the renal parenchyma; collecting tubule epithelial cells had nuclear inclusions consistent with BK infection, confirmed by immunolabeling and EM. Neither had evidence of C4d positivity in the interstitial capillaries. Electron microscopy identified concomitant viral infections in both subjects consistent with the morphology and size of WNV, which was subsequently confirmed by serology.

**Figure 1:**
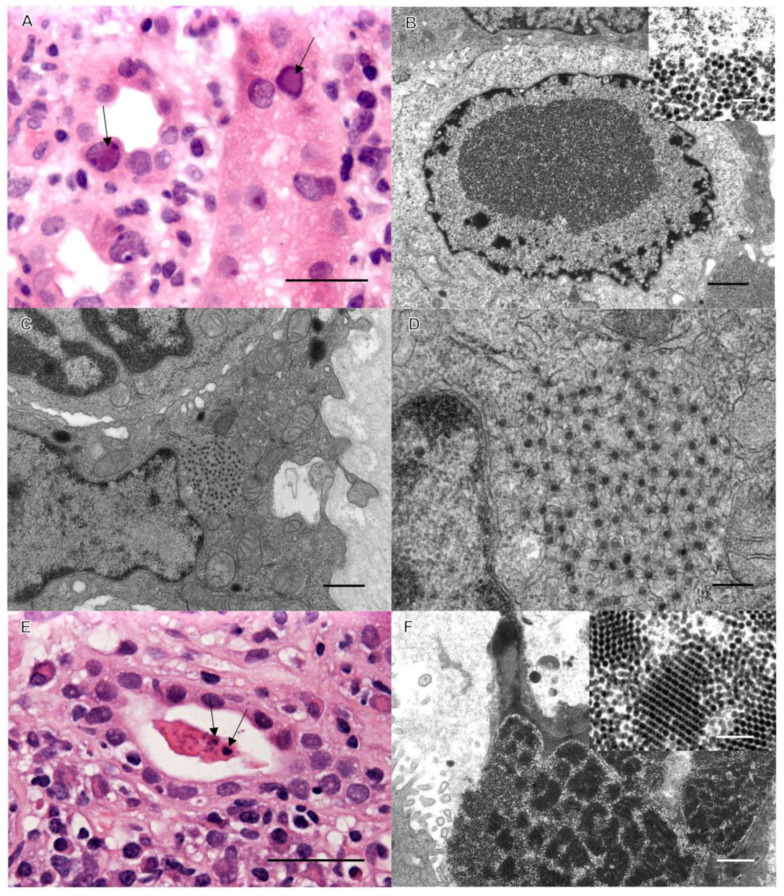
Renal cortex with viral inclusions. (**A**) Hematoxylin and eosin-stained image of classic nuclear inclusions of BK virus (arrows). Bar = 50 µm. (**B**) Ultrastructural image of intranuclear BK virions (bar = 1 µm); inset of 40–45 nm virions (bar = 0.1 µm). (**C**,**D**) EM demonstrates 50 nm intracytoplasmic viral inclusions consistent with WNV (bars: C = 1 µm, D = 0.5 µm). (**E**) Light microscopic image of an unusual collecting tubule cast with irregular densities (arrows). Bar = 50 µm. (**F**) These unusual casts consist of aggregates of WNV. Bar = 200 nm.

Case 1: Background. A 37-year-old male with end-stage renal disease (ESRD) secondary to Goodpasture syndrome received a deceased donor kidney transplant. His post-transplant status was excellent for 12 months, with blood urea nitrogen (BUN) and creatinine stable within normal ranges at 14 mg/dL and 1.12 mg/dL, respectively. His immunosuppression consisted of (Myfortic; also known as the generic drug CellCept) 360 mg of mycophenolate twice a day and 3 mg of tacrolimus twice a day. During a routine check-up, his creatinine and serum titers of BK virus were found to be elevated, and an assessment biopsy of his allograft was performed. Severe interstitial inflammation was observed, and immunohistochemistry (IHC) revealed that most of the lymphocytic infiltrates were CD3-positive; CD20 demonstrated moderately positive mononuclear cell aggregates, and CD19 and CD68 were occasionally positive. C4d was negative in interstitial capillaries, negating humoral rejection. Renal collecting tubules had prominent nuclear changes consistent with BK virus infection (Figure 1). Tubulitis was minimal, and the Banff score was scored zero (0) as the inflammatory cell infiltrates were deemed secondary to the BK virus infection. An ultrastructural investigation confirmed prominent BK activation, but a concomitant cytoplasmic viral infection was identified. While BK is a DNA virus (non-enveloped, icosahedral particles 40–45 nm in diameter) and replicates in epithelial cell nuclei, the concomitant virus was spherical, measuring approximately 50 nm in diameter, and was morphologically consistent with Flaviviridae (Figure 1). These virions were also being shed, forming large crystalline aggregates in collecting tubule lumina, consistent with an active infection (Figure 1). As above, an elevated serum level of BK virus was detected prior to biopsy. Serology was performed to confirm the ultrastructural observations suggestive of WNV, with WNV IgG elevated to 1.87 IU via an enzyme-linked immunosorbent assay (ELISA) and BK virus DNA elevated to 2,230,512 copies/mL via a polymerase chain reaction (PCR).

To ascertain whether the donor kidney could have harbored WNV, we contacted the health departments for the incidence of infected mosquitoes regionally near the donor’s residence for two years prior to death. During this time, WNV had not been isolated by county health departments. We also contacted the Ohio Department of Health to determine the incidence of WNV in humans, including blood donors, and found that there were 65 cases; none were in northwest Ohio. Although it is possible that the donor organ had a reservoir of WNV prior to transplantation, we were unable to identify specifically the source of the infection.

**Figure 2:**
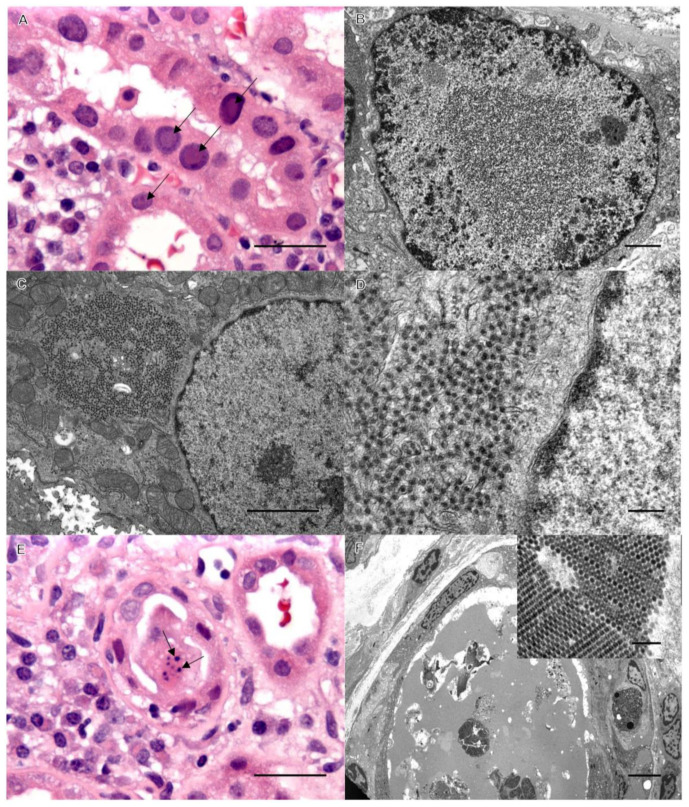
Classic viral inclusions of BK polyoma virus by light microscopy in panel (**A**) (arrows), bar = 50 µm, and by EM in panel (**B**). Bar = 1 µm. Panels (**C**–**F**) demonstrate identical WNV inclusions in case 2, as in case 1. Bars: C = 1 µm, D = 0.5 1 µm, E = 50 1 µm, and F = 20 1 µm (inset bar = 200 nm).

Case 2: Background. A 61-year-old male with ESRD secondary to polycystic kidney disease received a living unrelated kidney transplantation. His immunosuppression consisted of 1000 mg of myfortic twice daily and 1.5/1 mg of tacrolimus AM/PM. Due to a history of prostate cancer, myfortic was weaned, and he was started on 0.75 mg of everolimus twice a day His post-transplant status was stable for 5 months when his BUN, creatinine, and serum titers of BK virus were found to be elevated, and an assessment biopsy of his allograft was performed. Severe BK viral changes with inflammation and early transplant glomerulopathy were observed. An ultrastructural investigation identified collecting tubule cells with concomitant viral populations (Figure 2). Serology confirmed WNV IgG elevated to 2.23 IU by an ELISA, serum BK virus DNA elevated to 6,704,948 copies/mL by a PCR, and urine BK virus RNA elevated to 1,103,992,532 copies/mL by a PCR. Neither the donor nor the recipient was tested for WNV or BKV prior to transplant. The recipient did have elevated titers of WNV to confirm the EM diagnosis. Therefore, it is not possible to ascertain whether the patient had WNV prior to transplant.

## 4. Discussion

Herpes simplex virus, varicella zoster virus, Epstein–Barr virus, cytomegalovirus, hepatitis B virus, BK polyomavirus, and adenovirus are known viral infections in kidney transplant patients [17] (Table 1). Others include human immunodeficiency virus (HIV), hepatitis C, and West Nile virus [18].

BK virus is a double-stranded non-enveloped circular DNA virus. It is considered ubiquitous in humans, with seropositivity rates approaching 90% of the population [2,19]. Reactivation rates in kidney transplant recipients vary between 10% and 60% [1,2,20]. The clinical manifestation of BKV infection in the immunocompromised patient is specific to the urogenital tract and may include interstitial nephritis, cystitis, and/or ureteral stenosis [1]. The median time to develop BKV disease in the renal allograft is approximately 9 to 14 months, and the most common clinical manifestation is allograft dysfunction mimicking rejection [1,2]. Therefore, allograft biopsy should be performed to determine rejection versus viral infection for appropriate therapeutic intervention. BK-infected epithelial cells show enlarged nuclei with basophilic or amphophilic intranuclear viral inclusions (Figure 1 and Figure 2).

A transplant recipient’s immune characteristics as well as post-transplant immunosuppressive drugs, such as tacrolimus and mycophenolate mofetil, are considered the greatest risk factors for BK viral nephritis onset [21]. It is generally accepted that if the allograft recipient develops tissue-invasive nephropathy, graft outcome tends to be poor [17,22]. Both of our patients were being immunosuppressed with tacrolimus, mycophenolate, everolimus, and prednisone. Dosages of these agents were reduced, and renal function for both patients significantly improved.

West Nile virus is a mosquito-borne Flavivirus first isolated in the West Nile region of Uganda in 1937 [10,23]. The most common arthropod species involved in the transmission of WNV in the United States include Culex pipiens (northern house mosquito) and Culex quinquefasciatus (southern house mosquito) [10]. The virus was endemic and limited to Africa, the Middle East, West and Central Asia, and the Mediterranean regions [21]. Early epidemics affected rural populations, usually manifesting with systemic involvement and a few cases of severe neurological disease [10]. West Nile virus was detected in New York City in 1999 during an outbreak that resulted in 62 patients with encephalitis and 7 deaths [10]. It is now endemic to some regions of the United States, where it causes annual seasonal outbreaks [23,24].

West Nile virus particles are spherical, enveloped, approximately 50 nm in diameter, and have icosahedral symmetry. The WNV genome consists of a positive-sense single-stranded RNA that produces mature viral proteins via the proteolytic processing of a single polyprotein. These include three viral structural proteins (capsid, premembrane/membrane, and envelope) and seven nonstructural proteins (viral protease, NTPase, RNA helicase, and RNA-dependent RNA polymerase) [10]. As an RNA virus, it replicates in the cytoplasm of infected cells, in contrast to BK virus.

West Nile virus is maintained by cycling between many species of mosquitoes, their preferred vectors, and more than 300 species of birds, their naturally amplifying hosts. Humans and other mammals, especially horses, are incidental hosts with low viremic levels and do not play a role in the transmission cycle [10]. An estimated 70–80% of human WNV infections are asymptomatic; however, most symptomatic patients experience acute systemic febrile illness. Most WNV infections are acquired through bites from infected mosquitoes, but the virus can also be transmitted via the transfusion of infected blood products or solid organ transplantation [13,25].

There are a number of published case reports/series presenting documentation of WNV infections in transplanted organs in the United States (Table 2) [11,12,23,24]. In a study consisting of sixteen solid organ transplant recipients, twelve were infected; encephalitis developed in nine, and four of those nine died [25]. It was recently suggested that organ procurement organizations should consider screening for WNV prior to the transplantation of solid organs due to the significant health risks associated with the virus [26]. Transmission via tissue transplantation (i.e., skin, muscle, or connective tissues) has not been reported, and the potential risk for transmission by this route is unknown [25].

To our knowledge, this is the first report of concomitant WNV and BK viral infections in post-renal-transplant patients. Concomitant viral infections in renal allografts have been reported, including a case of cytomegalovirus and BK polyomavirus [27,28] and other cases of viral coinfections in liver transplants with Hepatitis B, C, and D viruses and Human Immunodeficiency Virus [29].

**Table 2:** Examples of previously reported cases of West Nile virus in allograft kidneys.

Clinical Presentation	Light Microscopy	Electron Microscopy	Ultimate Outcome
76-year-old male with meningoencephalitis [30]			Recovered
42-year-old male with meningoencephalitis [30]			Improved
38-year-old male with meningoencephalitis [31]	Immunohistochemical staining of brain tissue showed ample flavivirus antigens		Died
62-year-old male with encephalitis [32]	Encephalitis with mononuclear inflammation, microglial nodules, neuronal loss, and necrosis		Died
38-year-old male with meningoencephalitis [33]			Died
51-year-old male with encephalitis [34]			Improved
59-year-old female with encephalitis [34]			Died
14-year-old female with meningoencephalitis [35]			Recovered
58-year-old female with meningoencephalitis [14]	Renal biopsy: diabetic nodular glomerulosclerosis and mild interstitial infiltrate	Virion-like structures in the lumen of the renal tubules	Improved
63-year-old male with encephalitis [16]			Improved

Both of our patients were managed the same. Modulation of their immunosuppressive drugs, including a 50% reduction in mycophenolate and maintaining their prednisone, has increased renal function and reduced both viral titers for both; they are doing quite well without further flare-ups. Both are being monitored for mental status and neurological issues.

## 5. Conclusions

It is well established that transplant recipients receiving immunosuppressive drugs may be at risk for the reactivation of latent BK and CMV infections. It has also been established that WNV is harbored in the kidney, and it has been reported that it can be fatal in the immunocompromised patient. It is extremely important to consider that the reactivation of BK virus in a renal transplant recipient could mask a reactivation of West Nile virus. Our findings in two renal allograft recipients of concomitant BK and WNV suggest consideration of screening for WNV in deceased and living donors prior to transplantation in endemic regions of WNV-infected mosquitoes.

## Figures and Tables

**Table 1:** Viral infections in the allograft kidney.

Virus	Type	Replication	Diameter (nm)
West Nile virus	RNA	Cytoplasm	50
BK Polyomavirus	DNA	Nucleus	40–44
Adenoviridae	DNA	Nucleus	90–100
Cytomegalovirus	DNA	Nucleus	150–200
Epstein–Barr	DNA	Nucleus	120–150
Herpes	DNA	Nucleus	100–110
Varicella zoster	DNA	Nucleus	150–200
Hepatitis B	DNA	Nucleus	42

## Data Availability

The data presented in this study are available on request from the corresponding author. The data are not publicly available due to The United States Act of Congress Health Insurance Portability and Accountability Act of 1996 (HIPPA) regulations regarding personal health information.
